# Delayed Recurrence of Chromophobe Renal Cell Carcinoma Presenting as Metastatic Duodenal Ulcer

**DOI:** 10.7759/cureus.9154

**Published:** 2020-07-12

**Authors:** Nikita Jain, Anchit Bharat, Dipesh Ludhwani, Karam Khaddour, Thomas Weyburn

**Affiliations:** 1 Internal Medicine, Chicago Medical School, Rosalind Franklin University of Medicine and Science, McHenry, USA; 2 Internal Medicine, Indiana University Health Ball Memorial Hospital, Muncie, USA; 3 Internal Medicine, Rosalind Franklin University of Medicine and Science, North Chicago, USA; 4 Internal Medicine, Chicago Medical School, North Chicago, USA; 5 Internal Medicine, Chicago Medical School, Rosalind Franklin University, McHenry, USA; 6 Hematology and Oncology, Advocate Health Care, Crystal Lake, USA

**Keywords:** renal cell carcinoma, non clear cell renal carcinoma, duodenal ulcer, upper gastro-intestinal bleed, delayed recurrence, lymph node dissection, surveillance, chromophobe renal carcinoma

## Abstract

Renal cancers are one of the common causes of cancer-related morbidity and mortality worldwide. Most primary cases are localized at presentation and are treated with partial or radical nephrectomy with curative intent. However, renal cell carcinoma (RCC) is known for its potential recurrence, sometimes several years after initial management. Many of these recurrent cases commonly metastasize to the liver, kidney, or bone and herald a poor prognosis. We present a case study of nonclear cell RCC, which recurred 33 years after nephrectomy and masqueraded as a duodenal ulcer -- an extremely rare site for metastasis. This is unique as it describes a presentation only sparingly documented in the medical literature and highlights a more extended period of recurrence than currently reported. Moreover, our patient’s tumor was chromophobe cell variety, a rare sub-type of nonclear cell RCC, which to our knowledge has never been known to cause duodenal metastasis. Studies have implicated a prognostic role of lymph node involvement at the time of initial diagnosis to predict future recurrence. This case is a drop in the mighty ocean to prompt further investigation on the utility of life-long surveillance protocols and further research evaluating the role of lymph node dissection in preventing such recurrences and high mortality.

## Introduction

The incidence of renal cancers is projected to reach 73,750 new cases in 2020 [1]. Renal cell carcinoma (RCC) is classified broadly into two categories -- clear cell and nonclear cell RCC, with nonclear cell sub-types being the rarer variant. Nonclear cell RCC is further subclassified into papillary carcinoma, chromophobe carcinoma, oncocytoma, collecting duct carcinoma, and RCC unclassified [2]. Most primary cases are localized (65%) at initial presentation and are treated with partial or radical nephrectomy with curative intent [1]. However, relapse is common and can occasionally occur multiple decades after initial treatment [1, 3-4]. The estimated five-year survival rate is 92.6%. Conversely, among recurrent cases, up to 51% are metastatic and herald a poor prognosis, with overall survival as low as four months in high-risk patients [5-6]. Common sites for metastases in late recurrent RCC include the lung (36.4%), kidney (25%), and bone (13.6%) [7]. Metastasis to the gastrointestinal tract is rare, duodenum being the least commonly involved segment, with no documented reports of chromophobe cell RCC spreading to this site [8]. We discuss the case of a 73-year-old male who presented with a duodenal ulcer masquerading a relapsed chromophobe cell RCC, 33 years after nephrectomy. To our knowledge, this is a first of its kind presentation of chromophobe cell carcinoma, which is considered much less aggressive than clear cell RCC, with lower rates of recurrence.

## Case presentation

**Figure 1:**
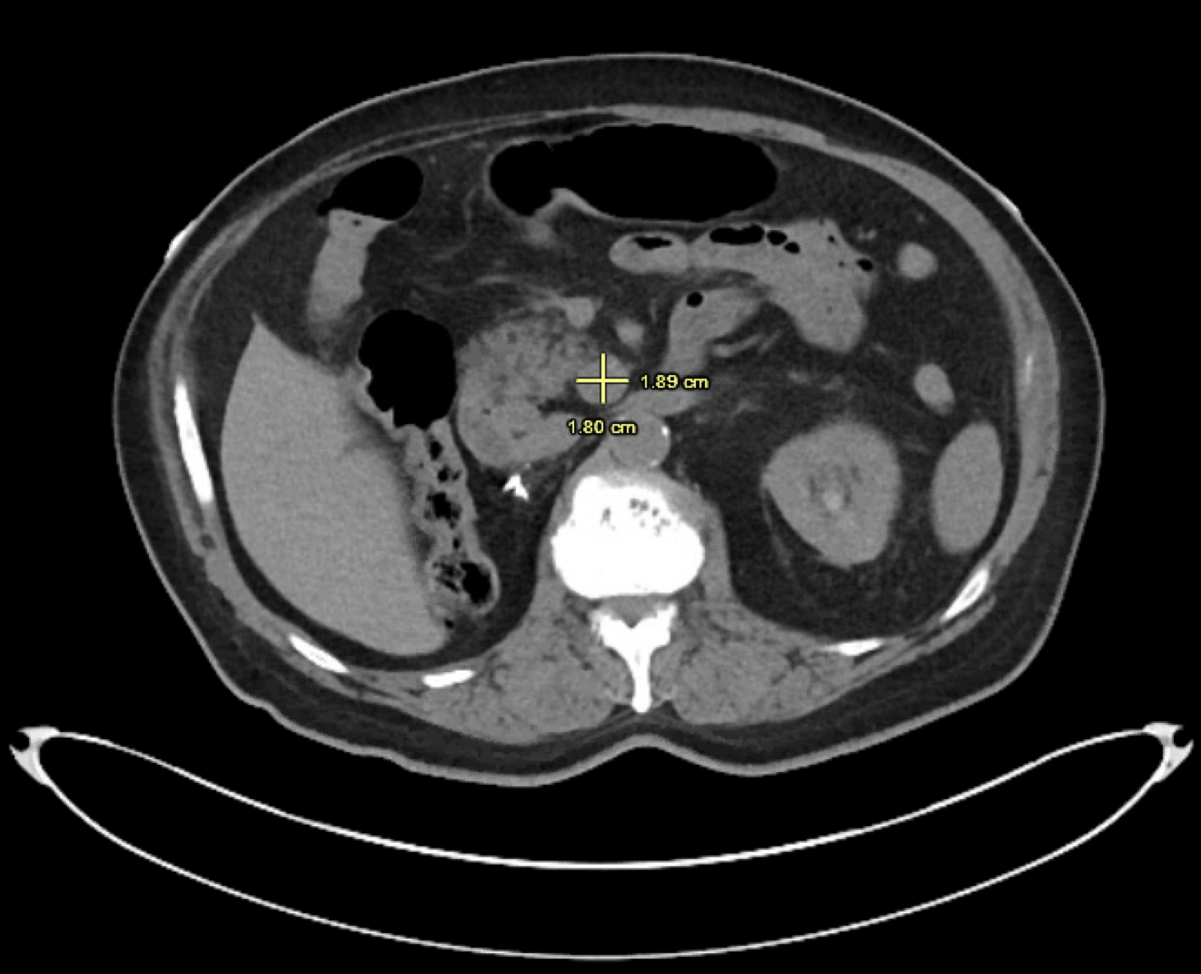
CTAP showing enlarged LN measuring 1.8 cm x 1.9 cm at the head of pancreas (dimensions marked in yellow). CTAP, computed tomography abdomen and pelvis; LN, lymph node

**Figure 2:**
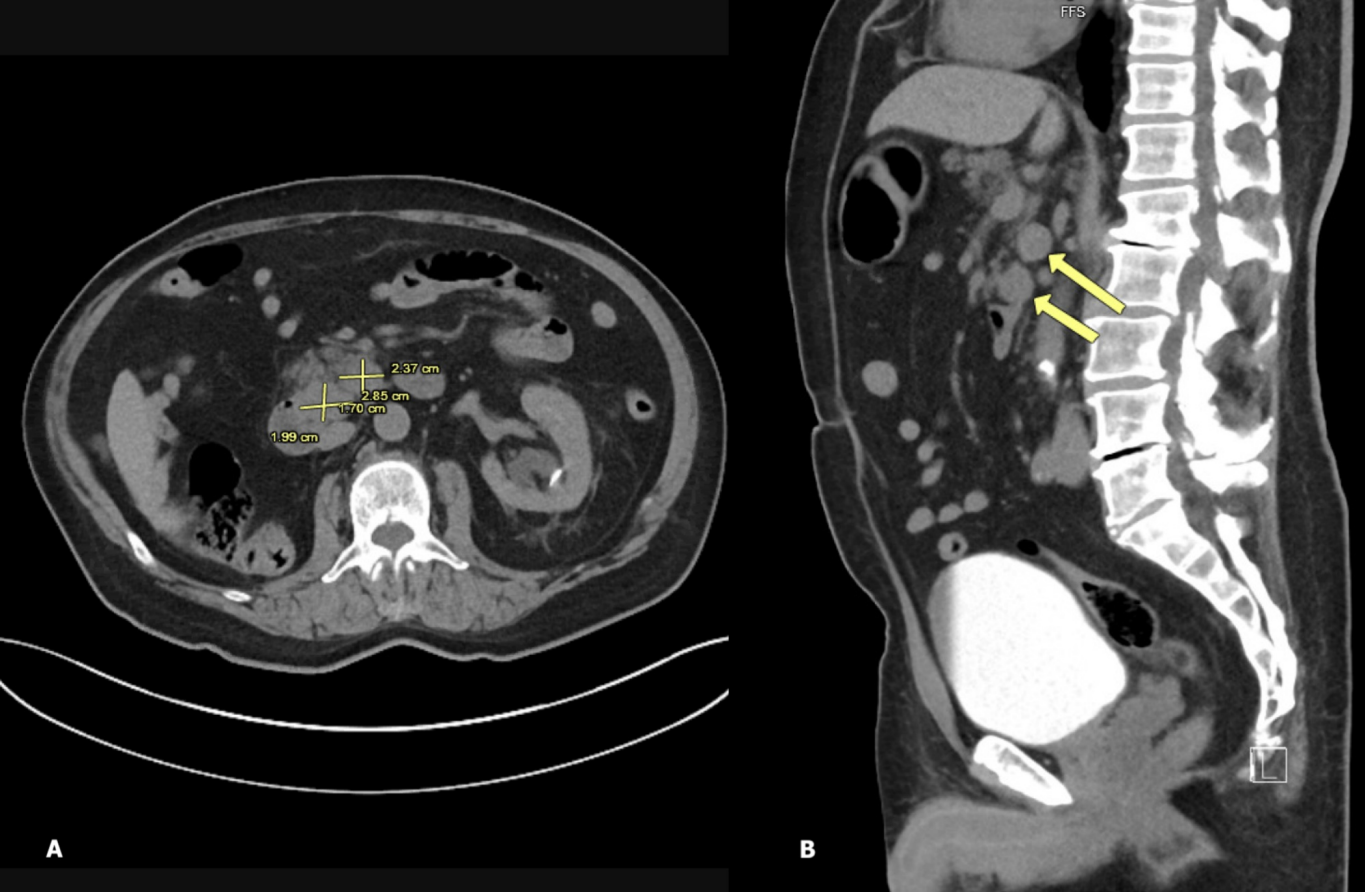
CTAP showing several enlarged LNs in pancreaticoduodenal region – one measuring 2.4 cm x 2.9 cm and another 2.0 cm x 1.7 cm in (A) transverse section (dimensions marked in yellow) and (B) sagittal section (arrows). CTAP, computed tomography abdomen and pelvis; LN, lymph node

**Figure 3:**
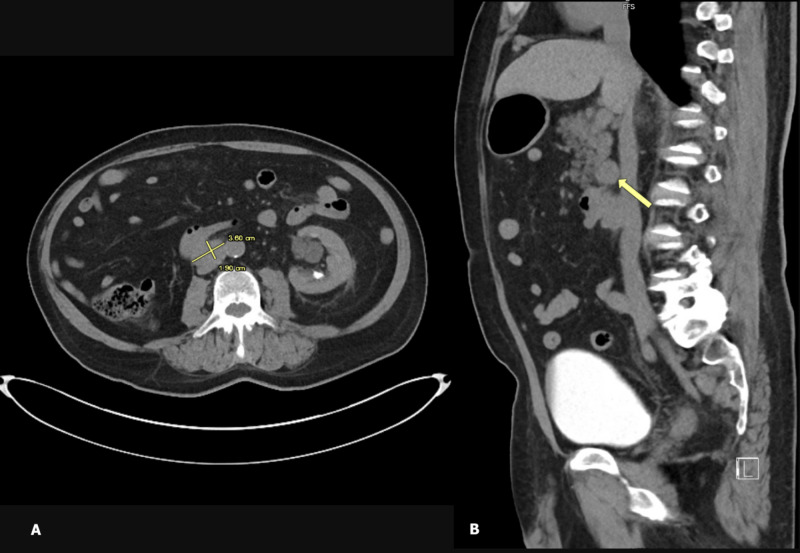
CTAP showing an enlarged LN anterior to the inferior vena cava – measuring 3.6 cm x 1.9 cm in largest dimension on (A) transverse section (dimensions marked in yellow) and (B) sagittal section (arrow). CTAP, computed tomography abdomen and pelvis; LN, lymph node

**Figure 4:**
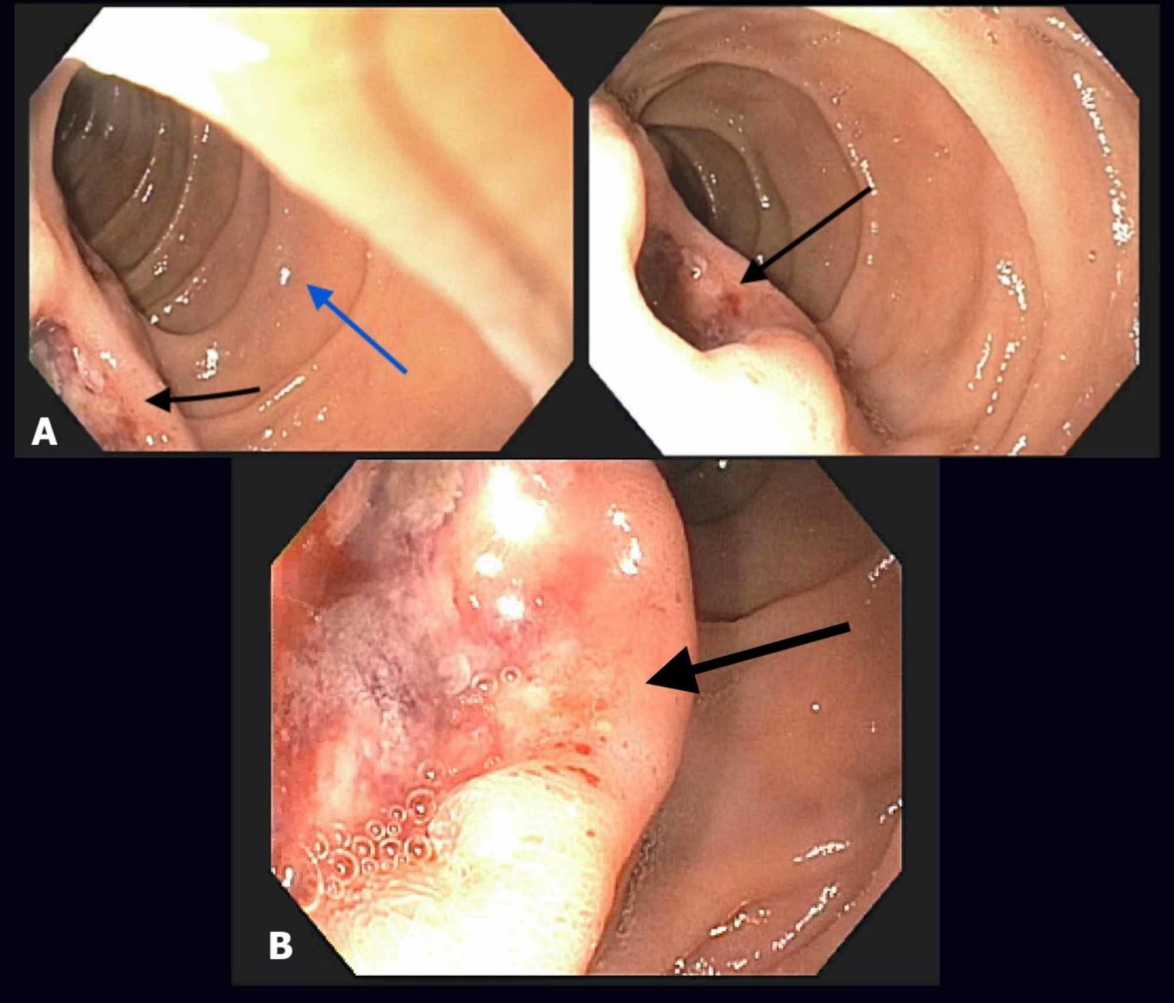
EGD showing a segment of duodenum. (A) A deep ulcer in the duodenal papilla with irregular margins (black arrows) and relatively normal appearance of second part of duodenum (blue arrow) is seen. (B) Enlarged view of the ulcer with a friable and malignant appearance (arrow). EGD, esophagogastroduodenoscopy

**Figure 5:**
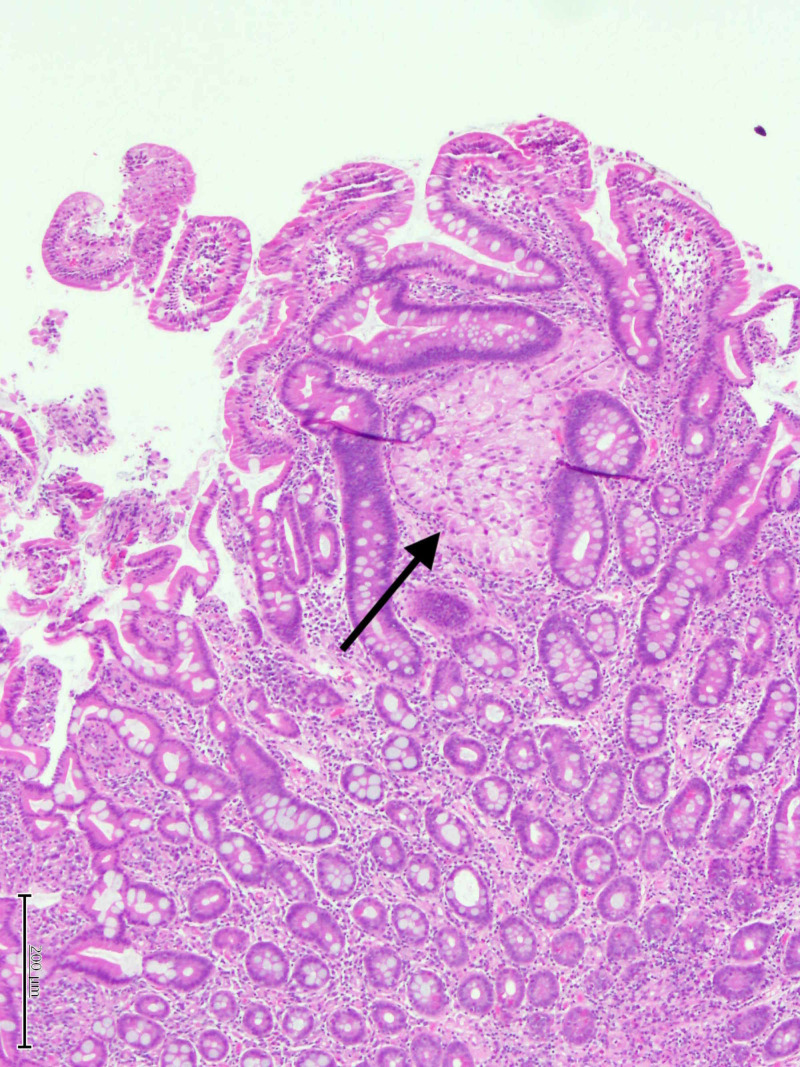
Hematoxylin and Eosin stain showing a focus of metastatic chromophobe RCC involving laminal propria of small bowel mucosa (arrow). RCC, renal cell carcinoma

**Figure 6:**
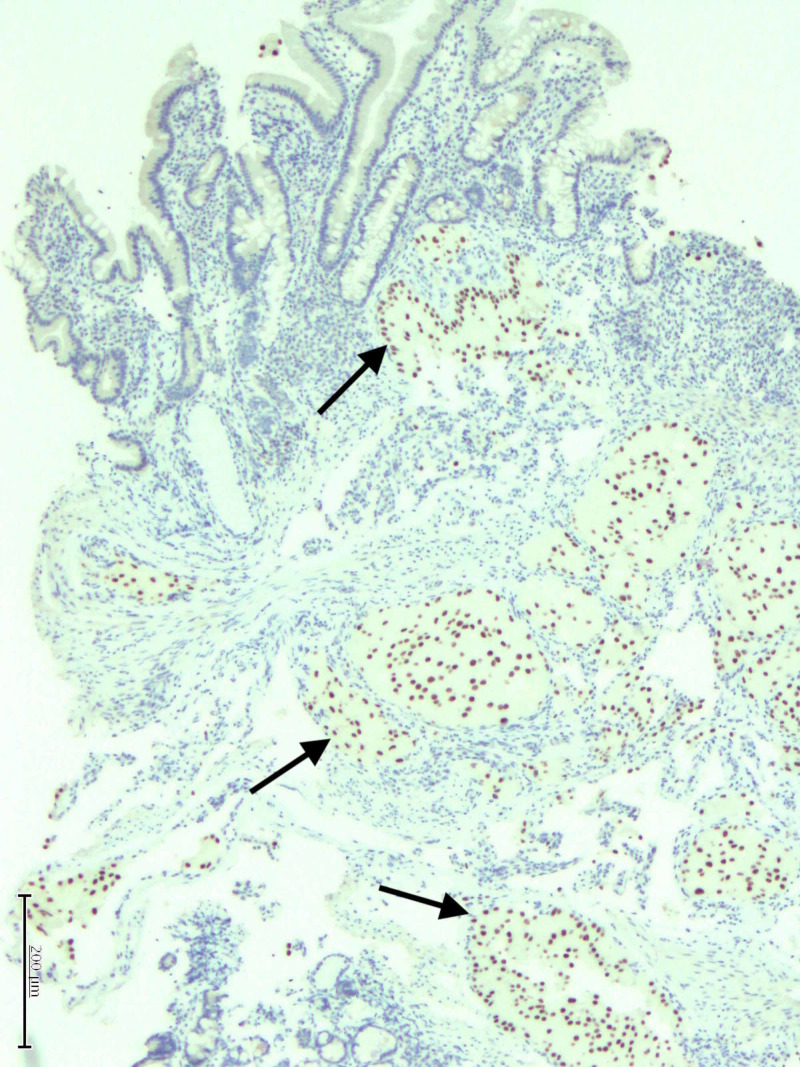
Metastatic RCC demonstrating strong nuclear PAX-8 reactivity by immunohistochemistry as indicated by arrows. RCC, renal cell carcinoma

A 73-year-old male with a past medical history of RCC, which was treated with right-sided radical nephrectomy 33 years prior, presented with abrupt onset moderate to severe right upper quadrant abdominal pain, radiating to the back. Associated symptoms included nausea and nonbloody vomiting. The patient denied any exposure to contaminated food, sick contacts, pharmacological agents like nonsteroidal anti-inflammatory drugs, corticosteroids, toxins, or alcohol. A review of systems revealed minor unintentional weight loss. Physical examination revealed a blood pressure of 208/95 mmHg, otherwise normal vital signs and epigastric tenderness. Laboratory evaluation was remarkable for microcytic anemia (hemoglobin 7.2 g/dL, mean corpuscular volume 72.2 fl). Iron studies showed low serum iron levels (15 ug/dL), low transferrin saturation (3%), and an elevated total iron-binding capacity (TIBC) (515 ug/dL). Stool guaiac was negative. CT scan of the abdomen and pelvis revealed several enlarged lymph nodes (LNs) in pancreaticoduodenal, para-aortic, and pre-caval regions, without the involvement of liver, pancreas, duodenum, or kidney (Figures 1-3). Considering the possibility of occult gastrointestinal bleeding, esophagogastroduodenoscopy (EGD) was performed, which revealed two deep, friable, malignant appearing ulcers in major duodenal papilla and a normal-appearing second portion of the duodenum (Figure 4). Biopsy from the ulcer margins showed small intestinal mucosa with scattered deposits of metastatic tumor cells with abundant pale cytoplasm, prominent cell borders, round nuclei, and prominent nucleoli (Figure 5). Immunohistochemistry showed strong positivity for PAX-8 and cytokeratin 7 and was negative for CD 10 and vimentin (Figure 6). This was consistent with a metastatic chromophobe RCC. Follow-up staging included a whole-body bone scan and MRI of the brain, which were negative for metastatic disease. The patient received supportive management for acute pain and iron deficiency anemia with outpatient follow-up care established with oncology. Due to extensive LN involvement, the patient was deemed to be a poor surgical candidate and was commenced on a combination of immune checkpoint inhibitors.

 

## Discussion

With the help of this case, we aim to grow awareness about duodenum as a potential metastatic site for RCC and describe its unusual presentation as a duodenal ulcer with occult gastrointestinal bleeding. Among the few cases documented in medical literature, duodenal involvement is only seen in co-existence with other distant metastasis like the liver and pancreas. Isolated metastasis to the duodenum is still sparse. Moreover, such a peculiar presentation remains undocumented in patients with chromophobe RCC. Known for their notorious tendency to relapse, RCC requires stringent surveillance after nephrectomy. American Urological Association recommends risk-based surveillance for up to five years and, thereafter based on clinical suspicion [9]. Yet, RCC might have a delayed recurrence, which can surpass the surveillance interval. This remains the most novel feature of our case study, as we bring to light a longer interval of relapse (33 years) than currently known. In light of this, a 10-year surveillance protocol has been suggested by some authors [10]. Our case provides evidence for the potential of a relatively indolent histologic type of RCC (chromophobe cell carcinoma) to cause a delayed and aggressive recurrence. Due to the unpredictable natural history and delayed recurrence of RCC, further research on the benefits and risks of longer surveillance is warranted. Previous studies have implicated a quiescent nature of tumor cells lying dormant in LNs to be a possible mechanism behind delayed recurrence of RCC. Such tumor cells are too small to be detected at initial diagnosis, but continually grow to evolve into clinically detectable recurrence [7, 11]. As such, regional LN involvement at primary diagnosis has been shown to predict recurrence after nephrectomy in several studies, with an inverse correlation between pathological tumor grade and interval of recurrence [12-14]. For instance, less aggressive tumors tend to recur after a longer lag interval. This is in alignment with the indolent nature of chromophobe carcinoma, which may explain its metastatic recurrence after a prolonged interval. Additionally, our patient exhibited metastatic involvement of LNs along typical lymphatic drainage of the right kidney (pre-caval, para-aortic LNs) and duodenum (pancreaticoduodenal LNs). This might favor a lymphatic route of spread from the renal bed to the duodenum (Figures 1-3). In accordance with this presentation and available current evidence, the role of LN dissection, if performed concurrently with initial nephrectomy, is an area for investigation, to whether it could prevent such recurrences. Current guidelines regarding LN dissection remain controversial [15]. Randomized controlled clinical trials involving LN mapping and sentinel LN biopsy might allow a better understanding of the pattern of lymphatic spread in RCC and may help establish strategies to prevent its recurrence.

## Conclusions

We propose that duodenum is a potential metastatic site for RCC, especially in relapsed cases, and may present as a duodenal ulcer with occult gastrointestinal bleeding. Although more common with clear cell renal carcinoma, this may occur in its indolent counterpart as well -- chromophobe cell carcinoma. The role of LN dissection to potentially prevent such recurrences is discussed, to prompt future randomized controlled trials exploring this dimension of the management of RCC.
